# Long noncoding RNA ILF3-AS1 aggravates papillary thyroid carcinoma progression via regulating the miR-4306/PLAGL2 axis

**DOI:** 10.1186/s12935-021-01950-8

**Published:** 2021-06-27

**Authors:** Zhaohui Zeng, Qiangfeng Teng, Jinhong Xiao

**Affiliations:** 1 Department of Nuclear Medicine, Hunan Provincial People’s Hospital/The First Affiliated Hospital of Hunan Normal University, Changsha, 410005 Hunan China; 2 Department of Nuclear Medicine, The First Affiliated Hospital of Guangxi Medical University, No. 6, Shuangyong Road, Nanning, 530021 Guangxi China; 3 Department of Laboratory, Hunan Provincial People’s Hospital/The First Affiliated Hospital of Hunan Normal University, Changsha, 410005 Hunan China

**Keywords:** Papillary thyroid carcinoma, ILF3-AS1, MiR-4306, PLAGL2

## Abstract

**Background:**

It have been proven that long non-coding RNAs (lncRNAs) serve as regulators in carcinogenesis. Interleukin enhancer binding factor 3 antisense RNA 1 (ILF3-AS1) has been illuminated as a prognostic factor in some cancers. Nevertheless, its expression pattern and possible functions in papillary thyroid carcinoma (PTC) have not been studied.

**Methods:**

The expression of ILF3-AS1 was measured by RT-qPCR and ISH. Colony formation assay and EdU assay were used to probe cell proliferation. TUNEL assay was used for analysis of cell apoptosis. Immunofluorescence and western blot were conducted to evaluate the expression change of E-cadherin and N-cadherin. The RNA interaction was demonstrated by mechanism experiments, including pull down assay and dual luciferase reporter assay.

**Results:**

ILF3-AS1 expression was evidently upregulated in PTC cell lines. ILF3-AS1 knockdown restrained the proliferation, migration and invasion of PTC cells. Mechanical investigation revealed that miR-4306 could interact with ILF3-AS1. PLAGL2 was a downstream target of miR-4306. The effects of ILF3-AS1 knockdown on the cellular processes were abrogated by miR-4306 downregulation or pleiomorphic adenoma gene-like 2 (PLAGL2) overexpression.

**Conclusion:**

ILF3-AS1 plays tumor-promoting role in PTC via targeting miR-4306/PLAGL2 axis.

**Supplementary Information:**

The online version contains supplementary material available at 10.1186/s12935-021-01950-8.

## Background

Among all the cancers from endocine organs, thyroid cancer (TC) is one of the most common carcinomas [1]. As the commonest histologic subtype of thyroid cancers, papillary thyroid cancer (PTC) accounts for approximately 85% of all thyroid cancer-related deaths [2]. Though PTC is a relatively moderate cancer with lower malignant degree, the incidence is increasing each year. Investigation of the molecular mechanism underlying PTC progression is of great significance for finding novel therapeutic targets.

The majority of human genome can be transcribed into RNA. Yet, only about 2% of human genome exhibit protein-coding capacity [3, 4]. Long non-coding RNAs (LncRNAs) refer to a class of transcripts with more than 200 bp, but their lack of open reading frames leads to the inability to encode proteins [5]. Recent years, lncRNAs have been reported in different human diseases due to their regulatory potentials in biological functions [6]. Interestingly, more and more lncRNAs have been found to regulate PTC progression via acting as miRNA sponge or competing endogenous RNA (ceRNA). For instance, lncRNA Gas5 sequesters miR-222-3p to inhibit proliferative ability of PTC cells [7]. LncRNA HOXA-AS2 as ceRNA facilitates the development of PTC cells by regulating miR-520c-3p to upregulate S100A4 [8].

ILF3-AS1 has been reported to be a putative biomarker for the diagnosis and treatment of prostate cancer [9]. ILF3-AS1 forms a positive feedback loop with ILF3 to facilitate melanoma cell proliferation and migration [10]. ILF3-AS1 has been found to be transcriptionally stimulated by SP1 and engaged in the ceRNA framework to promote osteosarcoma development [11]. ILF3-AS1 increases cell growth in retinoblastoma via miR-132-3p/SMAD2 axis [12]. However, it is unclear whether ILF3-AS1 exerted functions in thyroid cancer. Therefore, this study was aimed to monitor the possible functions of ILF3-AS1 in PTC progression.

MiR-4306 was firstly manifested to be an independent prognostic factor for disease recurrence of human papillomavirus (HPV)-negative head and neck squamous cell carcinoma [13]. Previous, exosomal miR-4306 was revealed to be possible diagnostic biomarker for PTC patients [14]. However, the functions and downstream target for miR-4306 remain to be unveiled in PTC.

Pleiomorphic adenoma gene-like 2 (PLAGL2) has been proved to activate Wnt/β-catenin signaling pathway and aggravated colorectal cancer development [15]. Furthermore, PLAGL2 was considered as a molecular target of miR-654-5p in ovarian cancer [16]. To date, the association between PLAGL2 and miR-4306 in PTC remains elusive.

The underlying mechanism of ILF3-AS1 in regulating PTC progression still remains to be explored, despite the fact that it has been found to participate into the modulation of multiple cancers. We aimed to figure out whether ILF3-AS1 could act as a therapeutic target to benefit the treatment of PTC patients. In conclusion, this study focused on the role of ILF3-AS1/miR-4306/PLAGL2 axis in PTC progression.

## Methods

### Cell lines

The normal human thyroid follicular epithelial cell line (Nthy-ori 3-1) was procured from European Collection of Authenticated Cell Cultures (ECACC, Porton Down, Salisbury, UK). Human thyroid cancer cell lines (IHH-4 and 8505C) were procured from Japanese Collection of Research Bioresources (JCRB) Cell Bank (Osaka, Japan), SW1736 cell line was procured from Cell Lines Service GmbH (CLS; Eppelheim, Baden Wurttemberg, Germany), TPC-1 cell line was procured from TOKU-E Company (Tokyo, Japan), and CGTH-W-3 cell line was procured from ATCC (Manassas, VA, USA). Apart from TPC-1 cell line cultured in Ham’s F-12 nutrient mixture (Gibco, Rockville, MD, USA), all of the cells lines were routinely grown in RPMI1640 medium (Gibco). 10% FBS (Gibco) and 1% antibiotics (Gibco) were acquired for cell culture purposes under 37 °C and 5% CO_2_.

### Total RNA isolation and RT-qPCR

Total RNAs were extracted by use of TRIzol Reagent (Invitrogen, Carlsbad CA, USA), followed by convertion into cDNA via PrimeScript Reverse Transcriptase Kit (Takara, Shiga, Japan). The quantitative assay was undertaken utilizing SYBR Green PCR Kit (Takara). The expression of RNA was calculated with 2^−ΔΔCt^ method and normalized to GAPDH, U6 or β-actin expression. The efficiency of primers were shown in Additional file 1.

### Western blot

Total proteins of IHH-4 and TPC-1 were isolated using RIPA lysis buffer. Then, SDS–polyacrylamide gel electrophoresisthe was performed to treat total proteins, followed by transferance onto the polyvinylidene fluoride (PVDF) membranes. Subsequently, the membranes were blocked with 5% non-fatted milk at room temperature for 2 h. Afterwards, the membranes were subjected to incubation with the primary antibodies against PLAGL2, E-cadherin, N-cadherin, Snail, and GAPDH overnight at 4 °C. After the elution, the membranes were incubated with the secondary non-specific antibodies for one hour at room temperature. The levels of proteins were then visualized and recorded. GAPDH served as the internal reference.

### Cell transfection

The shRNAs for ILF3-AS1 and control shRNAs were procured from Genechem (Shanghai, China). The sequence of PLAGL2 was inserted into pcDNA3.1 vector (Invitrogen) for overexpressing PLAGL2, the empty vector was used as negative control. Besides, the miR-4306 mimics, miR-4306 inhibitor and their negative control (NC mimics and NC inhibitor) were procured from GenePharma (Shanghai, China). IHH-4 and TPC-1 cells were seeded in 6-well plates (1 × 10^6^ cells/well) for transfection lasting 48 h, by use of Lipofectamine 3000 (Invitrogen). 10 ul mimics or inhibitor was used for transfection at the concentration of 100 nM.

### Colony formation assay

After transfection, IHH-4 and TPC-1 cells in 6-well plates were seeded at the density of 500 cells per well. Following culture for 2 weeks, the colonies were subjected to the treatment of 4% PFA for 30 min for fixation, and then stained for 5 min by using 0.5% crystal violet solution. Colonies were imaged and then counted.

### EdU assay

BeyoClick™ EdU Cell Proliferation Kit (Beyotime, Shanghai, China) was employed to carry out EdU assay in IHH-4 and TPC-1 cells with the utilization of Alexa Fluor 594. The transfected cells were fixed and permeabilized. Afterwards, the cells were stained by EdU medium, with the counterstaining of DAPI solution. After being washed in PBS, cells were observed by inverted microscope for analysis (Olympus, Tokyo, Japan).

### TUNEL assay

The transfected IHH-4 and TPC-1 cells were washed in PBS, and then fixation of cells was carried out using 4% PFA. TUNEL reagent (Merck KGaA, Darmstadt, Germany) was acquired to stain the apoptotic cells. Finally, the optical microscopy (Olympus) was applied for analysis.

### Flow cytometry analysis

In brief, collected PTC cells were washed by use of PBS. Then, cells were double-stained with Annexin V-labeled with 7AAD and PE purchased from BD Biosciences (San Jose, CA, USA). Apoptosis rate in different stages was measured by a Cytoflex flow cytometer (Beckman Coulter, CA, USA) and assessed by FlowJo software (Tree Star, USA).

### Wound healing assay

PTC cells were inoculated in 6-well plates when reached to 100% confluence. Cells were wounded with 1 mL pipette tips. Wound healing status were monitored and photographed at 0, 24 h after scratching.

### Transwell invasion assay

For invasion assay, PTC cells were incubated in the upper chamber of transwell chamber coated with Matrigel at the density of 2 × 10^4^ cells per well. The medium free of serum was added to the upper chamber, while the medium containing 10% FBS (Sigma-Aldrich, St. Louis, MO, USA) was supplemented to the lower chamber. Twenty four hours later, cells left on the upper surface of the membrane was removed. Cells in the lower chamber were stained with 0.5% crystal violet solution for 5 min and then were photographed under an inverted microscope (Leica, Germany).

### Immunofluorescence (IF) assay

Cells of IHH-4 and TPC-1 were cultured until adhered to slides, and then washed in PBS three times and fixed for 10 min. Cells in 5% BSA were prepared to incubate with the primary antibodies against E-cadherin and N-cadherin, and then with secondary antibodies. Following washing in PBS, cells were stained in DAPI solution and examined by Olympus confocal imaging system.

### In situ hybridization (ISH) assay

Enhanced Sensitive ISH Detection kit I (POD) (MK1030, Boster, USA) was used for ISH assay. After the fixation, the tumor tissues from the in vivo experiments were sliced and then treated with proteinase-K for incubation at 37 °C. After the hybridization of ILF3-AS1 probe, the slice was incubated with anti-DIG reagents. The signal of the probe was visualized using diaminobenzidine solution.

### Caspase-3 activity assay

Caspase 3 Activity Assay Kit (C1116, Beyotime, Shanghai, China) was used for the experiment. The total proteins of transfected IHH-4 and TPC-1 were obtained through using lysis buffer. Then the proteins were incubated with reaction buffer and caspase substrate. Caspase-3 activity was measured at wave length of 405 nm.

### In vivo study

Commercially obtained from the National Laboratory Animal Center (Beijing, China), Ten 6-week-old male BALB/c nude mice were fed in the SPF-grade laboratory. This in vivo experiment was approved by the Animal Research Ethics Committee of Hunan Provincial People’s Hospital. Ten nude mice were randomly and equally divided into two groups, and each mice was subcutaneously injected with TPC-1 cells into which sh-NC or sh-ILF3-AS1#1 was transfected. Twenty-eight hours later, tumor volume and weight were recorded every 4 days. Tumors were weighed after sacrificing mice.

### Subcellular fractionation

Cytoplasmic and nuclear RNA Isolation of IHH-4 and TPC-1 cells were undertaken by PARIS™ Kit (Invitrogen), as instructed by supplier. Cells were treated with cell fractionation buffer, followed by centrifugation. The isolated RNAs were detected in cell cytoplasm and cell nucleus using RT-qPCR.

### Fluorescence in situ hybridization (FISH)

IHH-4 and TPC-1 cells were fixed, and then air-dried for culturing with ILF3-AS1-specific FISH probe (Ribobio, Guangzhou, China) in line with user guide. After hybridization, cells were subjected to Hoechst staining and analyzed using Olympus microscope.

### RNA pull down assay

RNA interaction was assayed in IHH-4 and TPC-1 cells by Pierce Magnetic RNA–Protein Pull-Down Kit procured from Thermo Fisher Scientific (Waltham, MA, USA). The cell protein lysates were prepared for mixing with biotinylated ILF3-AS1 or miR-4306 probes, following the adding of streptavidin agarose magnetic beads. Relative RNA enrichment in mixture was assayed by RT-qPCR.

### Luciferase reporter assay

The ILF3-AS1 or PLAGL2 3′UTR fragments covering wild-type (WT) or mutant (Mut) miR-4306 binding sites were prepared for generating pmirGLO-ILF3-AS1-WT/Mut and pmirGLO-PLAGL2 3′UTR-WT. They were co-transfected into IHH-4 and TPC-1 cells with NC mimics or miR-4306 mimics for 48 h. The results were finally studied using luciferase reporter assay system acquired from Promega (Madison, WI, USA).

### RNA immunoprecipitation (RIP)

As instructed by manufacturer (Millipore, Bedford, MA, USA), RIP assay was undertaken by Magna RIP™ RNA-Binding Protein Immunoprecipitation Kit. After the lysis, cell lysates were incubated with control IgG antibody or human Ago2 antibody in magnetic beads for immunoprecipitation. RT-qPCR was finally used for analysis.

### Statistical analyses

Data from three separately conducted assays were presented as the mean ± SD with GraphPad Prism 7 (GraphPad Software, Inc., La Jolla, CA, USA). Student’s t-test was used to compare the difference between two groups, and one-way ANOVA to analyze the differences among more than two groups. The data obtained from experiments were defined to be statistically significant when p < 0.05.

## Results

### ILF3-AS1 is expressed at a high level in PTC cell lines

**Fig. 1:**
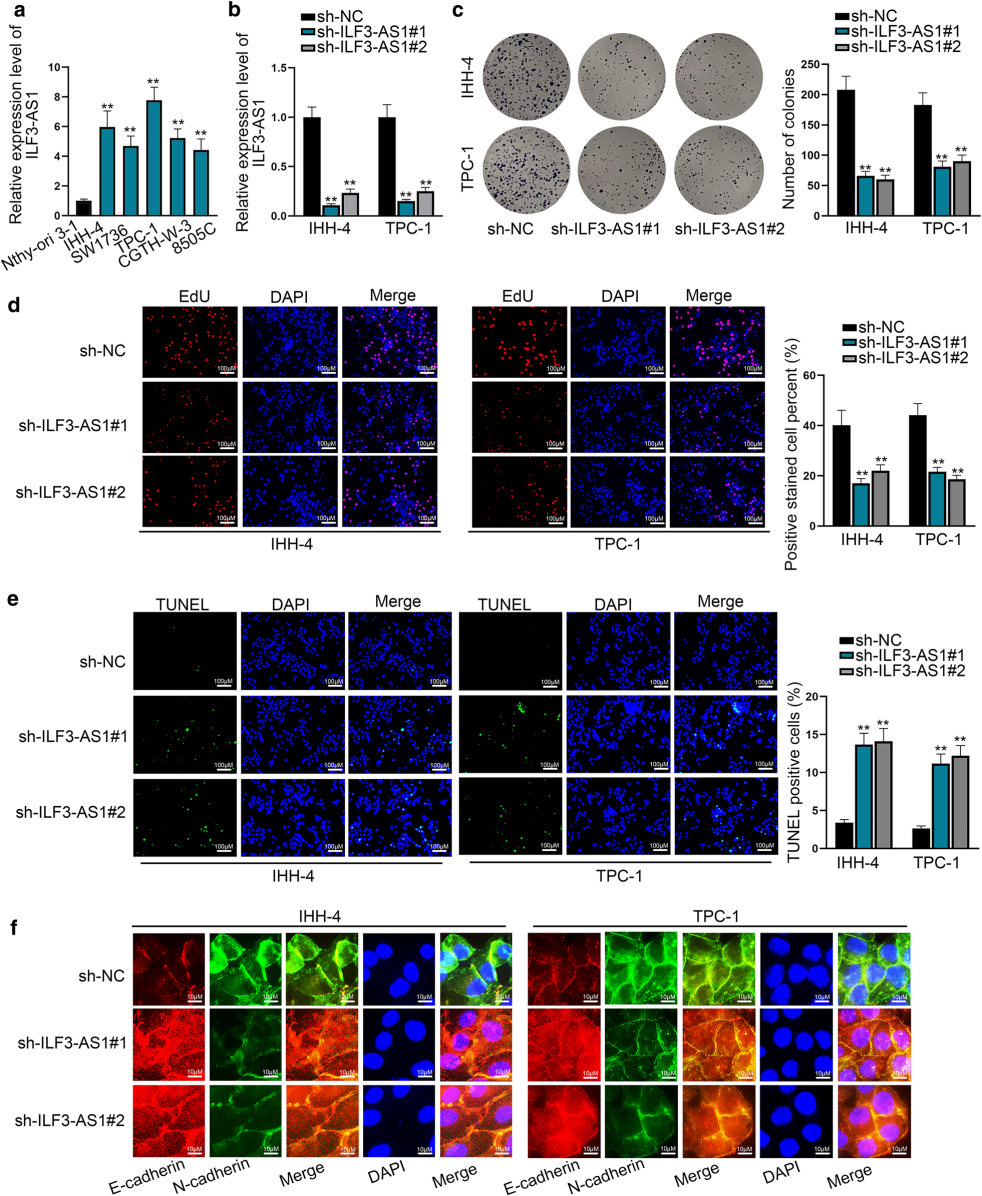
ILF3-AS1 is expressed at a high level in PTC cell lines. **a** The expression level of ILF3-AS1 in five PTC cell lines and one normal thyroid cell line was analyzed by RT-qPCR. **b** ILF3-AS1 expression was interfered by transfection of sh-ILF3-AS1#1/2 into IHH-4 and TPC-1 cells. **c**, **d** Colony formation assay and EdU assay were conducted to probe into the effects of sh-ILF3-AS1#1/2 on PTC cell proliferation. **e** TUNEL assay was used to evaluate the influence of sh-ILF3-AS1#1/2 on apoptosis. **f** IF assay explored the expressions of EMT markers after the ILF3-AS1 knockdown. ^**^P < 0.01

To explore whether ILF3-AS1 involved in the progression of PTC, we firstly checked its expression in five PTC cell lines (IHH-4, SW1736, TPC-1, CGTH-W-3, 8505C) and one normal thyroid cell line (Nthy-ori3-1). As a result, ILF3-AS1 expression was higher in PTC cell lines than that in the normal thyroid cell line (Fig. 1a). The data from TCGA (http://gepia2.cancer-pku.cn/#analysis) verified that ILF3-AS1 was upregulated in THCA tissues (Additional file 2: Figure S1A). The ends of ILF3-AS1 were identified by 3′ and 5′ RACE experiment (Additional file 2: Figure S1B). The functions of ILF3-AS1 in PTC were further determined by performing loss-of-function assays. In advance of functional assays, we used ILF3-AS1-specific shRNAs (sh-ILF3-AS1#1 and sh-ILF3-AS1#2) to silence ILF3-AS1 in IHH-4 and TPC-1 cells (Fig. 1b). ILF3-AS1 silencing resulted in the inhibition of cell proliferation as evidenced by the decrease of colonies and EdU-positive cells (Fig. 1c, d). Subsequently, we found that ILF3-AS1 silencing accelerated apoptosis in PTC cells as proved by the results of TUNEL assay, flow cytometry analysis and caspase 3 activity test (Fig. 1e and Additional file 2: Figure S1C-D). In addition, the capacities of PTC cells to invade and migrate were suppressed after the silencing of ILF3-AS1 as evidenced by wound healing and transwell assays (Additional file 2: Figure S1E-F). Finally, we monitored EMT process through performing IF assay and western blot analysis. The results showed that ILF3-AS1 silencing augmented the expression of E-cadherin, while lessening N-cadherin and Snail expression (Fig. 1f and Additional file 2: Figure S1G). In vivo experiments further indicated the oncogenic role of ILF3-AS1 in PTC. The tumor growth was inhibited after silencing of ILF3-AS1 (Additional file 3: Figure S2A–C). Moreover, ISH assay showed that the level of IFS-AS1 was markedly lowered in tumor tissues in sh-ILF3-AS1#1 group (Additional file 3: Figure S2D). Taken together, the data showed the up-regulation of ILF3-AS1 promotes PTC progression.

### ILF3-AS1 interacts with miR-4306 in PTC cells

**Fig. 2:**
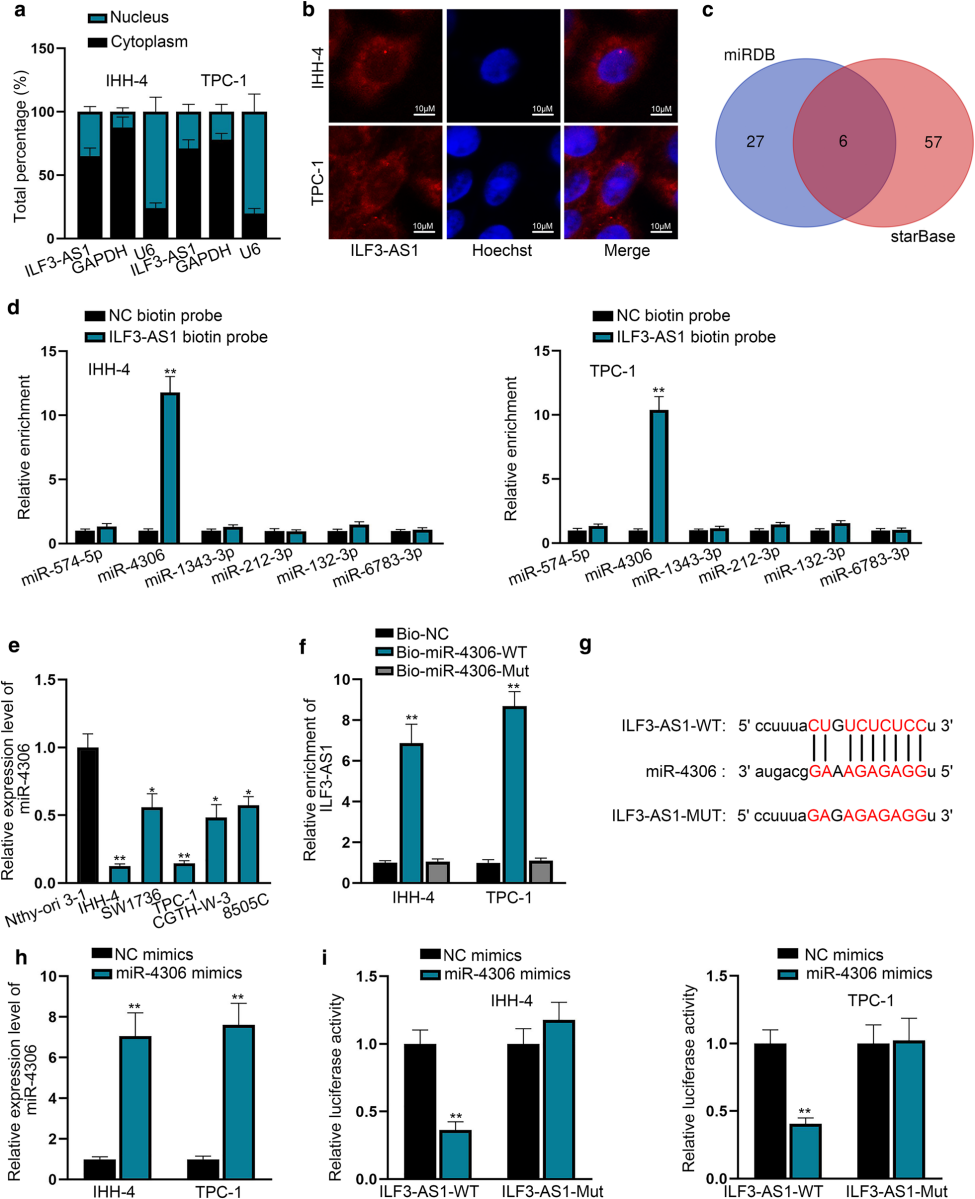
ILF3-AS1 interacts with miR-4306 in PTC cells. **a**, **b** The distribution of ILF3-AS1 in IHH-4 and TPC-1 cells was detected by subcellular fractionation assay and FISH assay. **c** Bioinformatics tool screened out 6 miRNAs that may have the ability to interact with ILF3-AS1. **d** RNA pull down assay was performed to test the interaction between 6 candidate miRNAs and ILF3-AS1. **e** The expression level of miR-4306 in PTC cell lines was assessed by RT-qPCR. **f** RNA pull down assay showed that ILF3-AS1 was pulled down abundantly by biotinylated miR-4306-WT. **g** The putative binding sites of miR-4306 with ILF3-AS1 were predicted. **h** The overexpressing efficacy of miR-4306 mimics was examined in IHH-4 and TPC-1 cells. **i** Dual Luciferase reporter assay was applied to prove the mutual interaction between ILF3-AS1 and miR-4306. ^*^P < 0.05, ^**^P < 0.01

To investigate the underlying molecular mechanism of ILF3-AS1 in PTC cells, we firstly identified its cellular distribution in PTC cells. Through nuclear and cytoplasmic fraction RNA and FISH assays, we identified that ILF3-AS1 was mainly located in the cytoplasmic fraction of IHH-4 and TPC-1 cells (Fig. 2a, b). Cytoplasmic lncRNAs have the ability to sponge miRNAs to modulate cancer development [17]. In this study, we predicted six miRNAs (Fig. 2c) potentially interacting with ILF3-AS1 by utilizing miRDB (http://mirdb.org/) and starbase bioinformatics tools (http://starbase.sysu.edu.cn/). To demonstrate whether ILF3-AS1 could interact with these six miRNAs, we conducted RNA pull down assay. It turned out that only miR-4306 was enriched in the complex pulled down by biotinylated ILF3-AS1 probe (Fig. 2d). Unlike ILF3-AS1, miR-4306 was lowly expressed in PTC cell lines (Fig. 2e). To further prove the interaction between miR-4306 and ILF3-AS1, we used bio-miR-4306-WT and bio-miR-4306-Mut to conduct RNA pull down assay. As shown in Fig. 2f, ILF3-AS1 was enriched in bio-4306-WT group but not in bio-miR-4306-Mut group (Fig. 2f). The putative binding sites between miR-4306 and ILF3-AS1 were obtained and illustrated in Fig. 2g. Then, we overexpressed miR-4306 (Fig. 2h) to perform dual luciferase reporter assay. It was uncovered that the luciferase activity of ILF3-AS1-WT group was obviously weakened by the upregulation of miR-4306 (Fig. 2i). Additionally, we silenced miR-4306 in PTC cells (Additional file 4: Figure S3A) for the implementation of dual luciferase reporter assay again. The results indicated that silencing of miR-4306 led to the reinforcement of luciferase activity of ILF3-AS1-WT group but had no significant effect on ILF3-AS1-Mut group (Additional file 4: Figure S3B). Collectively, we confirmed that ILF3-AS1 can interact with miR-4306.

### MiR-4306 suppresses PTC cell proliferation and migration

**Fig. 3:**
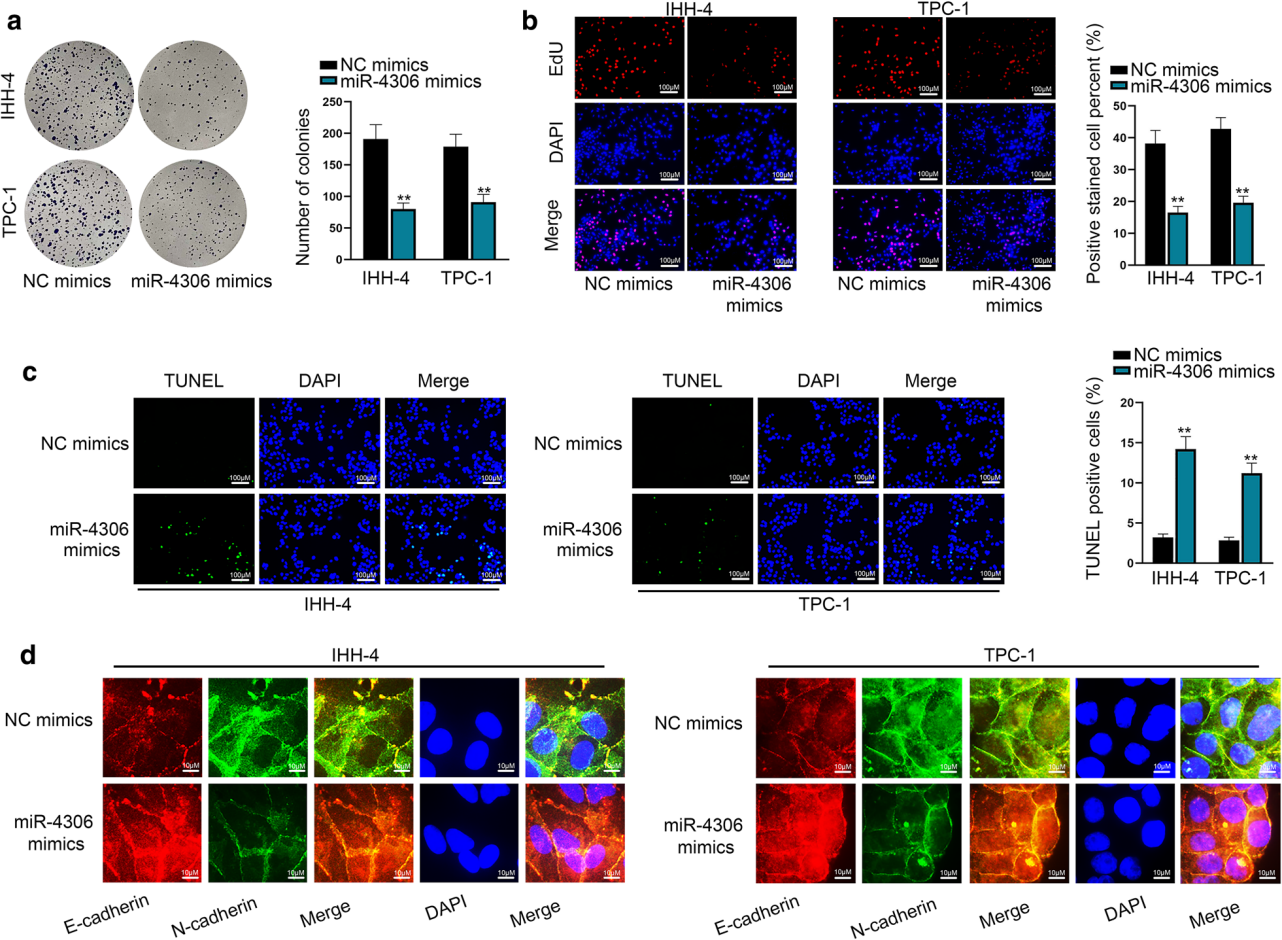
MiR-4306 suppresses PTC cell proliferation and migration. **a**, **b** The impacts of miR-4306 mimics on PTC cell proliferation were investigated through colony formation assay and EdU assay. **c** The effects of miR-4306 upregulation on PTC cell apoptosis were detected by TUNEL assay. **d** EMT process was observed in PTC cells with miR-4306 upregulation through IF assay. ^**^P < 0.01

Subsequently, gain-of-function assays were performed to demonstrate the effects of miR-4306 on PTC cellular functions. The decline of colonies and EdU-positive cells manifested that miR-4306 overexpression suppressed the proliferation ability of PTC cells (Fig. 3a, b). Moreover, the increased apoptosis induced by miR-4306 overexpression was observed through TUNEL assay, flow cytometry analysis and caspase 3 activity test (Fig. 3c and Additional file 5: Figure S4A-B). The inhibitory effects of miR-4306 mimics on the migratory and invasive capacities of PTC cells were also determined in accordance with the results of wound healing assay and transwell assay (Additional file 5: Figure S4C-D). At last, the increase of miR-4306 led to the increased E-cadherin level, and knocked down N-cadherin and Snail level (Fig. 3d and Additional file 5: Figure S4E), suggesting that miR-4306 could inhibit EMT process. Loss-of function assays were also conducted to prove the tumor-suppressive role of miR-4306 in PTC. As expected, knockdown of miR-4306 strengthened the abilities of PTC cell to proliferate, migrate and invade (Additional file 6: Figure S5A-D). These results indicated that miR-4306 plays a tumor-suppressive role in PTC.

### PLAGL2 is a downstream target of miR-4306

**Fig. 4:**
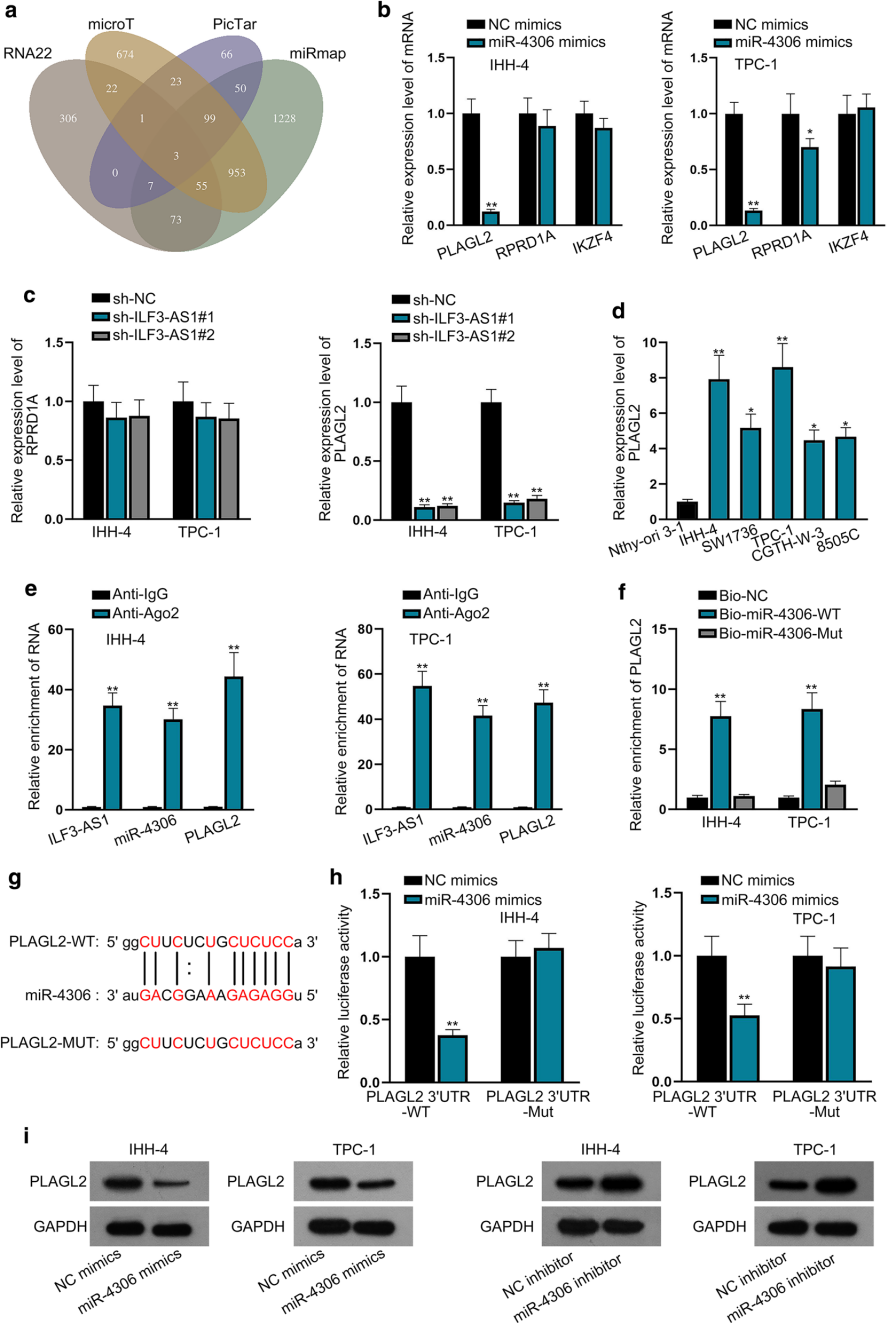
PLAGL2 is a downstream target of miR-4306. **a** Venn diagram showed 3 common mRNA candidates for miR-4306 predicted by four databases. **b**, **c** RT-qPCR assay probed into the effects of miR-4306 mimics or sh/ILF3-AS1#1/2 on the expression of candidate mRNAs. **d** The expression level of PLAGL2 in PTC cells was identified by RT-qPCR. **e** Ago2-RIP assay demonstrated the enrichment of ILF3-AS1, miR-4306 and PLAGL2 in anti-Ago2 group or anti-IgG group. **f** The interaction between miR-4306 and PLAGL2 was proven by RNA pull down assay. **g**, **h** The predicted binding sites between miR-4306 and PLAGL2 was shown and verified by dual luciferase reporter assay. **i** Western blot analysis was conducted to detect the protein level of PLAGL2 after the overexpression or knockdown of miR-4306. ^*^P < 0.05, ^**^P < 0.01

Next, the downstream target of miR-4306 was predicted and verified. Based on the prediction of four bioinformatics tools (RNA22, microT, PicTar and miRmap), 3 mRNAs (PLAGL2, RPRD1A, IKZF4) were found to be able to bind with miR-4306 (Fig. 4a). Among these three mRNAs, only PLAGL2 was significantly downregulated by miR-4306 upregulation or ILF3-AS1 silencing (Fig. 4b, c). Moreover, PLAGL2 was highly expressed in PTC cells (Fig. 4d). Thus, we chose PLAGL2 to be the candidate target gene. Data of Ago2-RIP assay demonstrated that ILF3-AS1, miR-4306 and PLAGL2 were co-precipitated by the anti-Ago2 (Fig. 4e), indicating that these three RNAs were all enriched in RNA-induced silencing complex (RISC). The interaction between miR-4306 and PLAGL2 was proven by RNA pull down assay (Fig. 4f). Then, the binding sites between miR-4306 and PLAGL2 were obtained (Fig. 4g). The undermined luciferase activity of PLAGL2-WT in miR-4306-overexpressed cells further indicated the interaction between miR-4306 and PLAGL2 (Fig. 4h). Finally, the results of western blot analysis showed that protein level of PLAGL2 was decreased after the overexpression of miR-4306, but was significantly increased by the silencing of miR-4306 (Fig. 4i). All these data indicated the conclusion that PLAGL2 is the molecular target of miR-4306.

### ILF3-AS1 expedites PTC progression via sponging miR-4306 to elevate PLAGL2 expression

**Fig. 5:**
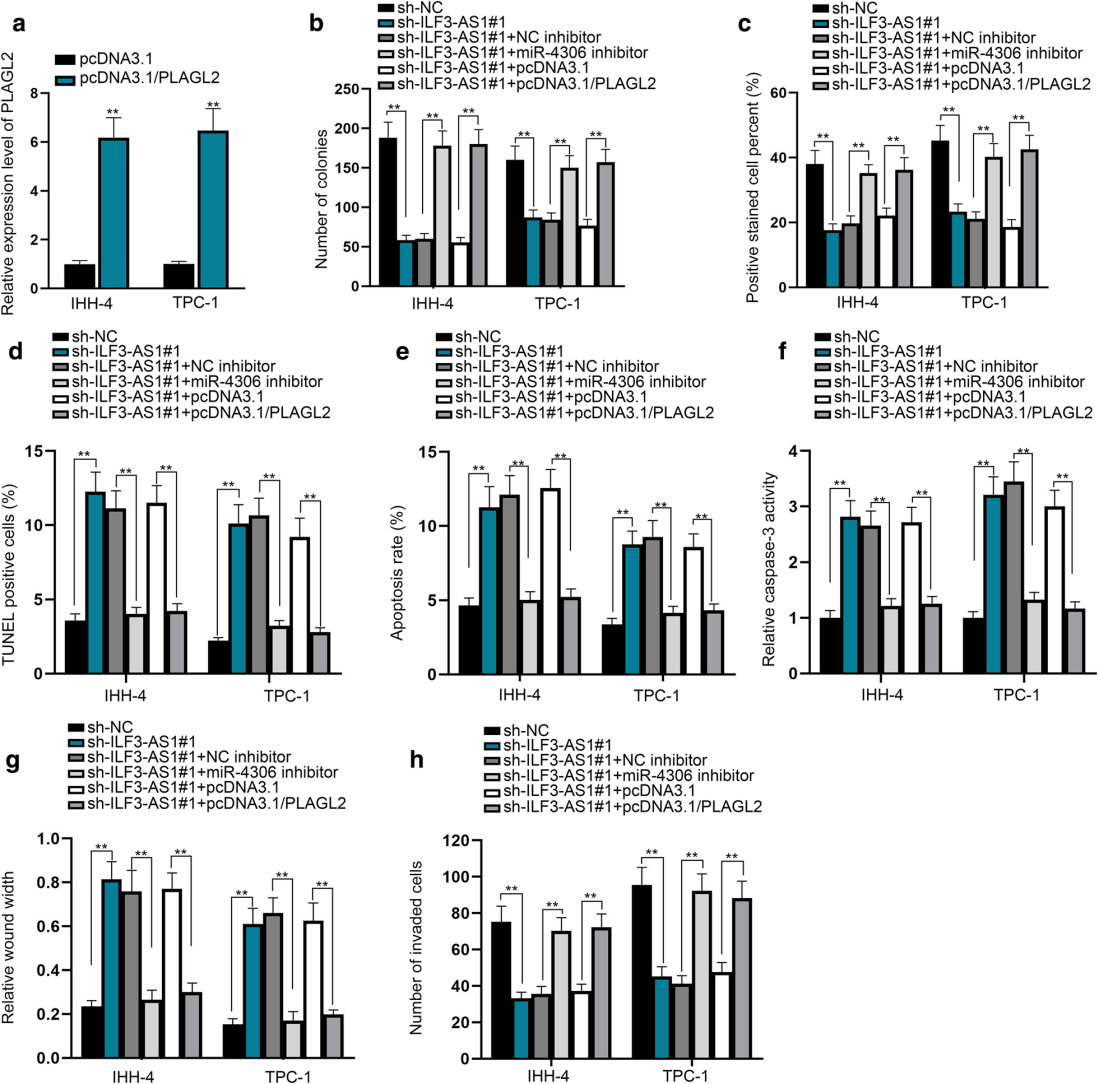
ILF3-AS1 expedites PTC progression via sponging miR-4306 to elevate PLAGL2 expression. **a** The expression of PLAGL2 was analyzed in response to pcDNA3.1/ PLAGL2. **b**, **c** The effects of miR-4306 and PLAGL2 on ILF3-AS1-mediated cell proliferation were demonstrated by colony formation assay and EdU assay. **d**–**f** The changes in apoptosis were observed in transfected PTC cells through TUNEL assay, flow cytometry analysis and caspase 3 activity detection. **g**, **h** The rescuing effects of miR-4306 inhibitor and pcDNA3.1/PLAGL2 on suppressed migratory and invasive capacities by sh-ILF3-AS1 were detected by wound healing assay and transwell assay. ^**^P < 0.01

Rescue assays were implemented to prove the role of ILF3-AS1/miR-4306/PLAGL2 regulatory axis in PTC progression. To begin with, we upregulated PLAGL2 expression in IHH-4 and TPC-1 cells (Fig. 5a). Cell proliferation ability suppressed by ILF3-AS1 silencing was restored by the inhibition of miR-4306 or the overexpression of PLAGL2, according to the results of colony formation and EdU assays (Fig. 5b, c). Based on the results of TUNEL assay, flow cytometry analysis and caspase 3 activity detection, we determined that the silenced ILF3-AS1-induced apoptosis was attenuated by the depression of miR-4306 or the reinforcement of PLAGL2 (Fig. 5d–f). Additionally, cell migration and invasion suppressed by ILF3-AS1 knockdown were recovered by miR-4306 silencing or PLAGL2 overexpression (Fig. 5g–h). Furthermore, the results of western blot analysis showed EMT suppressed by ILF3-AS1 silencing was countervailed by miR-4306 ablation or PLAGL2 overexpression, as verified by the expression of E-cadherin, N-cadherin and Snail (Additional file 6: Figure S5E). Collectively, ILF3-AS1 plays a regulatory role in PTC cell growth and migration through targeting miR-4306/PLAGL2 axis.

## Discussion

NcRNAs have been acknowledged as essential regulators in tumorigenesis [18, 19]. The role of lncRNAs as novel prognostic and diagnostic biomarkers in several human malignancies, including laryngeal cancer, have been revealed [20]. LncRNAs have been elaborated to play pivotal roles in gene modulation, including genomic imprinting and alternative splicing [21–23]. Over the past decade, multiple lncRNAs have been elucidated to regulate the development of malignancies. For example, Hai Liu et al. have discovered that lncRNA HNF1A-AS1 accelerates the progression of gastric cancer through modulating the expression of cell cycle-related proteins to affect cell cycle distribution [24]. LncRNA AWPPH strengthens cell proliferation, migration and autophagy in bladder cancer by down-regulating SMAD4 [25]. According to Shen et al. LINC00152 mediated the stabilization of PTEN to modulate PTC onset and advance [26]. Mechanistically, lncRNAs can act as ceRNAs to exert functions on human cancers. For instance, SNHG15 acts as a ceRNA to up-regulate YAP1 through interacting with miR-200a-3p, further leading to the promotion of PTC [27]. In our research, ILF3-AS1 was verified to be overexpressed in PTC cell lines. ILF3-AS1 has been previously reported as a tumor facilitator in melanoma [10], osteosarcoma [11] and nasopharyngeal carcinoma [28]. In this study, we uncovered that ILF3-AS1 silencing posed a negative impact on the proliferative, migratory and invasive abilities, while had a positive effect on PTC cell apoptosis. ILF3-AS1 depletion also affected E-cadherin and N-cadherin level, which revealed the crucial role of ILF3-AS1 in regulating the EMT process. Taken together, our exploration demonstrated that ILF3-AS1 promotes PTC progression.

MiRNAs have been illuminated to play a regulatory role in various cellular processes, such as proliferation, apoptosis, migration, autophagy and drug resistance [29–32]. Plentiful reports have validated the joint effect of lncRNAs and miRNAs in multiple types of malignancies. For instance, lncRNA XIST contributes to gastric cancer via cooperating with the miR-185/TGF-β1 axis [33]. In our study, miR-4306 has been predicted and proven as the downstream miRNA harboring the binding sites with ILF3-AS1. Moreover, miR-4306 was underexpressed in PTC cell lines, and it suppressed the malignant behaviors via targeting PLAGL2. Previously, PLAGL2 was validated to promote EMT process in colorectal cancer via regulating ZEB1 [34]. Here, we uncovered that PLAGL2 is involved in ILF3-AS1-mediated PTC cellular processes. Collectively, this study unmasked a novel ceRNA pathway in PTC progression, which may help to uncover a novel therapeutic target for PTC treatment.

## Conclusion

LncRNA ILF3-AS1 acted as an oncogene in PTC via targetingmiR-4306/PLAGL2 axis. This finding may provide a theoretical evidence for using ILF3-AS1 as a target in future treatment of PTC. However, lack of clinical study is a deficiency of our current study. Thus, we will further probe into the association of clinicopathological features of PTC patients with ILF3-AS1, miR-4306 and PLAGL2 in our future study.

## Supplementary Information

**Additional file 1. **Primer efficiency of primers used in experiments

**Additional file 2: Figure S1.** Knockdown of ILF3-AS1 accelerates cell apoptosis but suppresses cell migration and invasion. A. GEPIA detected the expression of ILF3-AS1 in THCA tissues. B. The ends of ILF3-AS1 were identified by 3′ and 5′ RACE experiment. C. Flow cytometry analysis evaluated the apoptosis in PTC cells with ILF3-AS1 silencing. D. Caspase 3 activity in ILF3-AS1-silenced PTC cells was tested. E. Wound healing assay was conducted to determine migratory ability of PTC cells after the ILF3-AS1 silencing. F. Transwell invasion assay was conducted in ILF3-AS1-downregulated PTC cells. G. Western blot assay evaluated the protein levels of EMT markers after the knockdown of ILF3-AS1. ^**^P < 0.01.

**Additional file 3: Figure S2.** ILF3-AS1 silencing lead to the inhibition on PTC cell growth in vivo. A. It showed the tumors in two groups of mice injected with PTC cells transfected with sh-NC or sh-ILF3-AS1#1. B-C. Tumor volume and tumor weight in two groups were shown. D. ILF3-AS1 expression in tumor tissues in two groups was detected by ISH assay. ^**^P < 0.01.

**Additional file 4: Figure S3** The interaction between miR-4306 and ILF3-AS1. A. Rt-qPCR assessed miR-4306 expression in two PTC cells after the silencing of miR-4306. B. Dual luciferase reporter assay indicated that silencing of miR-4306 led to the enhanced luciferase activity in ILF3-AS1-WT group. ^**^P < 0.01.

**Additional file 5: Figure S4** Upregulation of miR-4306 induces cell apoptosis and suppresses cell migration and invasion. A-B. The increased apoptosis induced by miR-4306 overexpression was observed through flow cytometry analysis and caspase 3 activity test. C-D. The inhibitory effects of miR-4306 mimics on the migration and invasion of PTC cells were also determined in accordance with the results of wound healing assay and transwell assay. E. Western blot assay was performed to detect the protein levels of EMT markers after the overexpression of miR-4306. ^**^P < 0.01.

**Additional file 6: Figure S5** miR-4306 inhibition facilitates PTC cell growth and migration. A-B. Colony formation and EdU assays probed into the effects of miR-4306 inhibition on PTC cell proliferation. C-D. The effects of miR-4306 silencing on the capacities of PTC cells to migrate and invade were also determined by wound healing assay and transwell assay. E. Western blot analysis was used to detect the protein levels of EMT markers in transfected IHH-4 and TPC-1 cells. ^**^P < 0.01.

## Data Availability

Not applicable.

## Abbreviations

lncRNAs: Long non-coding RNAs
PTC: Papillary thyroid carcinoma
3′ UTR: 3′ Untranslation region
TC: Thyroid cancer
PTC: Papillary thyroid cancer
ceRNA: Competing endogenous RNA
miRNAs: MicroRNAs
ILF3-AS1: Interleukin enhancer binding factor 3 antisense RNA 1
PLAGL2: Pleiomorphic adenoma gene-like 2
