# Supplementary material for: Long noncoding RNA ILF3-AS1 aggravates papillary thyroid carcinoma progression via regulating the miR-4306/PLAGL2 axis
Source: Cancer Cell Int. 2021 Jun 27;21:322. doi: 10.1186/s12935-021-01950-8 (PMC8237480; doi:10.1186/s12935-021-01950-8)
Supplement: Supplementary file 1 — Additional file 1. Primer efficiency of primers used in experiments [file 12935_2021_1950_MOESM1_ESM.docx]

| **Primer efficiency of primers used in experiments** | |
| --- | --- |
| primer | efficiency |
| sh-NC(for ILF3-AS1) | 95% |
| sh-ILF3-AS1#1 | 94% |
| sh-ILF3-AS1#2 | 95% |
| ILF3-AS1 | 96% |
| GAPDH | 96% |
| U6 | 95% |
| NC biotin probe | 97% |
| ILF3-AS1 biotin probe | 97% |
| miR-4306 | 96% |
| PLAGL2 | 97% |
| RPRD1A | 96% |
| IKZF4 | 96% |
