# Supplementary figures and images for: Long noncoding RNA ILF3-AS1 aggravates papillary thyroid carcinoma progression via regulating the miR-4306/PLAGL2 axis
Source: Cancer Cell Int. 2021 Jun 27;21:322. doi: 10.1186/s12935-021-01950-8 (PMC8237480; doi:10.1186/s12935-021-01950-8)

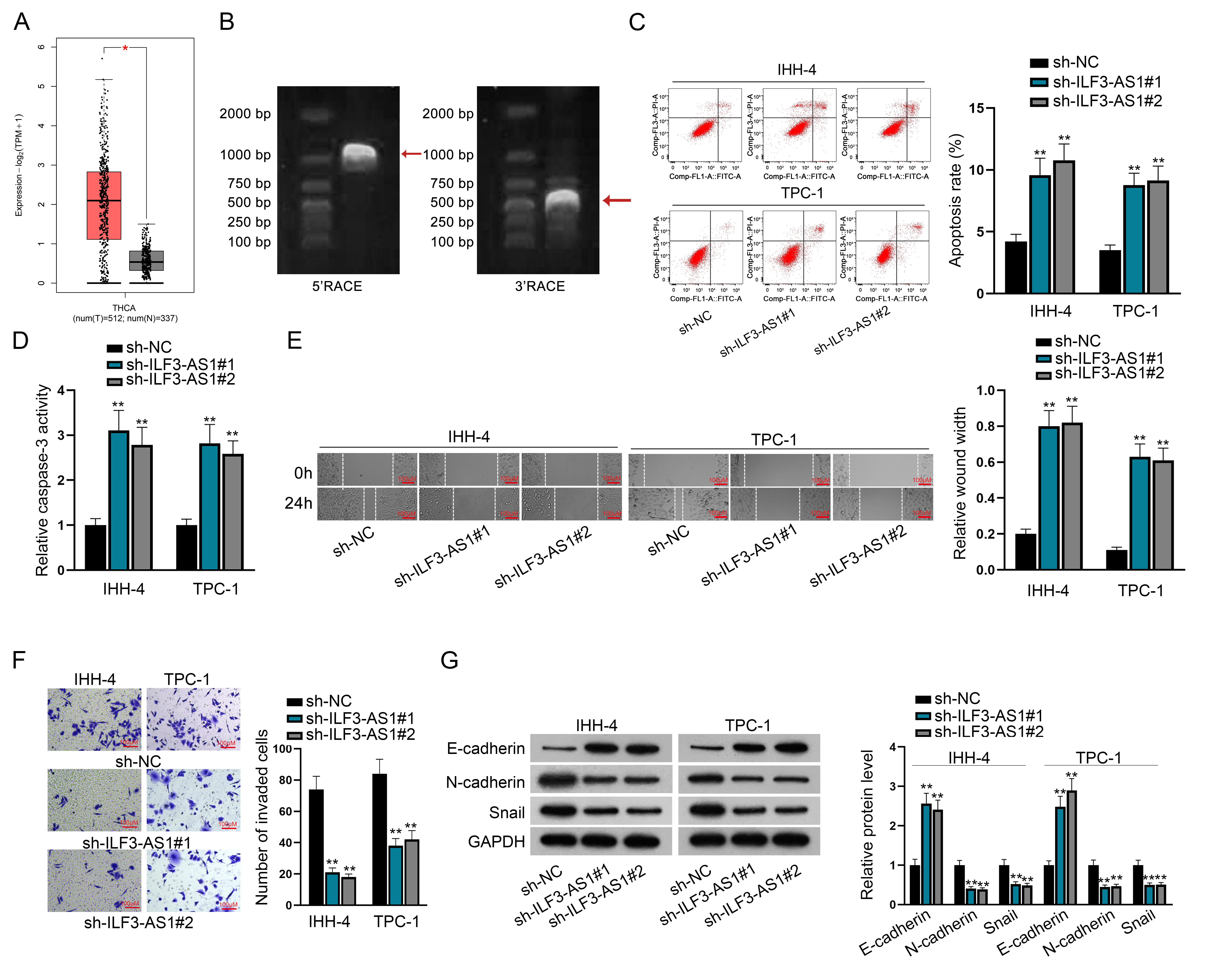

Supplement: Supplementary file 2 — Additional file 2: Figure S1. Knockdown of ILF3-AS1 accelerates cell apoptosis but suppresses cell migration and invasion. A. GEPIA detected the expression of ILF3-AS1 in THCA tissues. B. The ends of ILF3-AS1 were identified by 3′ and 5′ RACE experiment. C. Flow cytometry analysis evaluated the apoptosis in PTC cells with ILF3-AS1 silencing. D. Caspase 3 activity in ILF3-AS1-silenced PTC cells was tested. E. Wound healing assay was conducted to determine migratory ability of PTC cells after the ILF3-AS1 silencing. F. Transwell invasion assay was conducted in ILF3-AS1-downregulated PTC cells. G. Western blot assay evaluated the protein levels of EMT markers after the knockdown of ILF3-AS1. **P < 0.01. [file 12935_2021_1950_MOESM2_ESM.tif]

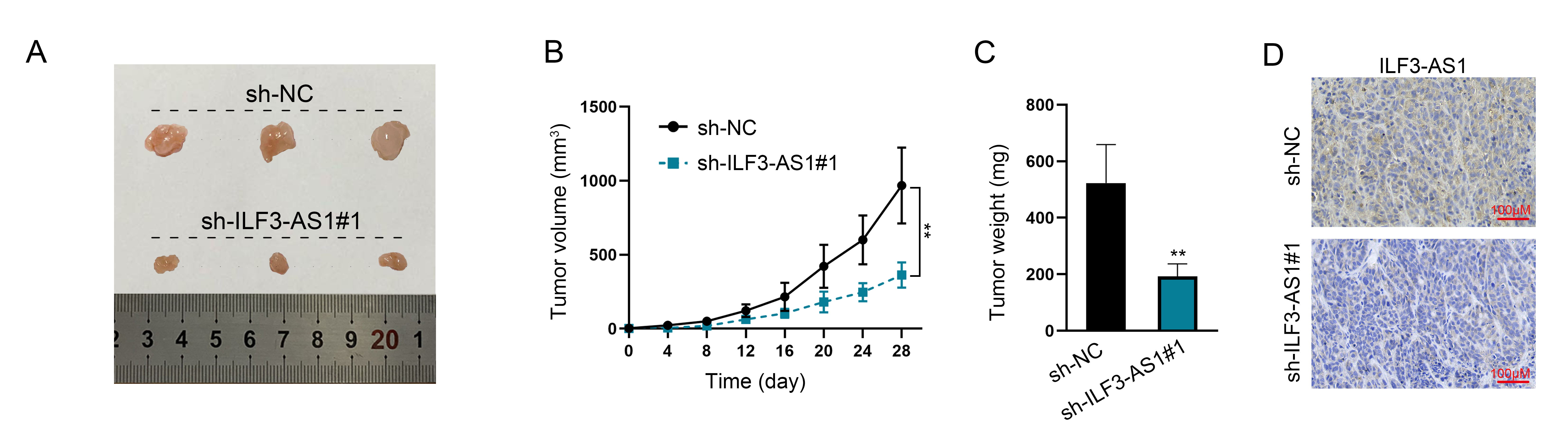

Supplement: Supplementary file 3 — Additional file 3: Figure S2. ILF3-AS1 silencing lead to the inhibition on PTC cell growth in vivo. A. It showed the tumors in two groups of mice injected with PTC cells transfected with sh-NC or sh-ILF3-AS1#1. B-C. Tumor volume and tumor weight in two groups were shown. D. ILF3-AS1 expression in tumor tissues in two groups was detected by ISH assay. **P < 0.01. [file 12935_2021_1950_MOESM3_ESM.tif]

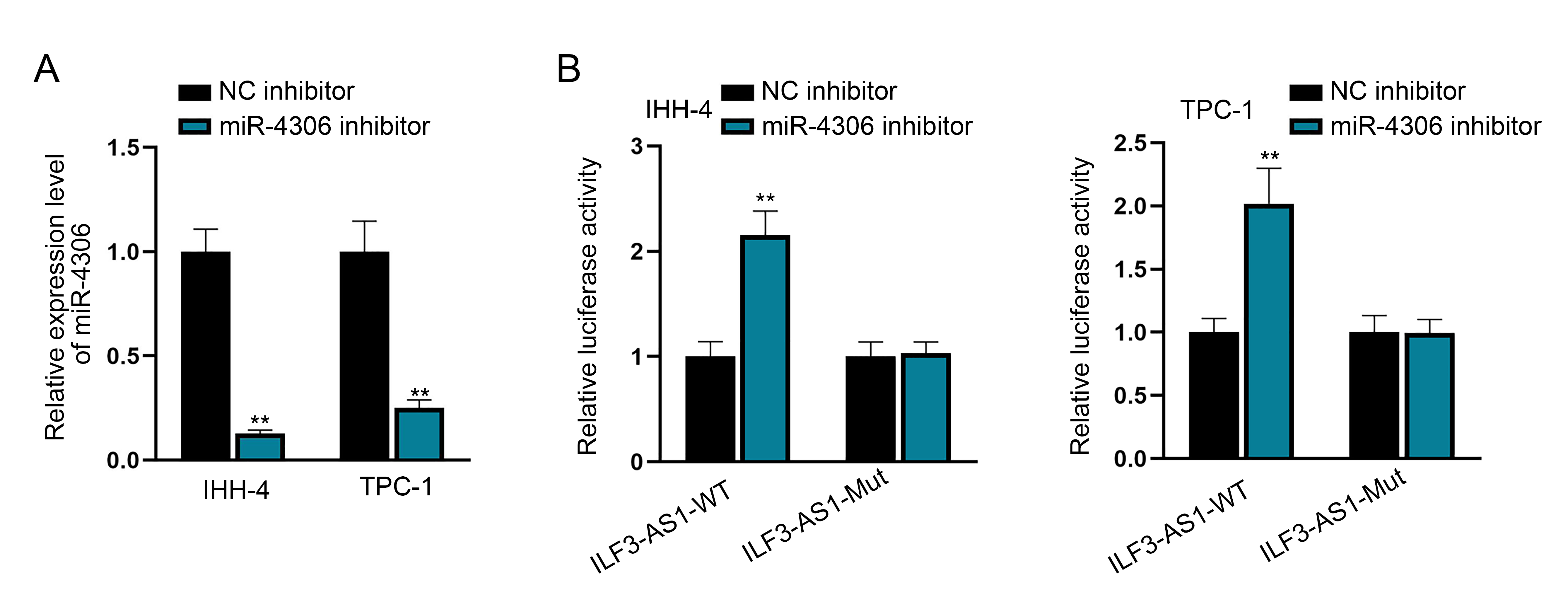

Supplement: Supplementary file 4 — Additional file 4: Figure S3 The interaction between miR-4306 and ILF3-AS1. A. Rt-qPCR assessed miR-4306 expression in two PTC cells after the silencing of miR-4306. B. Dual luciferase reporter assay indicated that silencing of miR-4306 led to the enhanced luciferase activity in ILF3-AS1-WT group. **P < 0.01. [file 12935_2021_1950_MOESM4_ESM.tif]

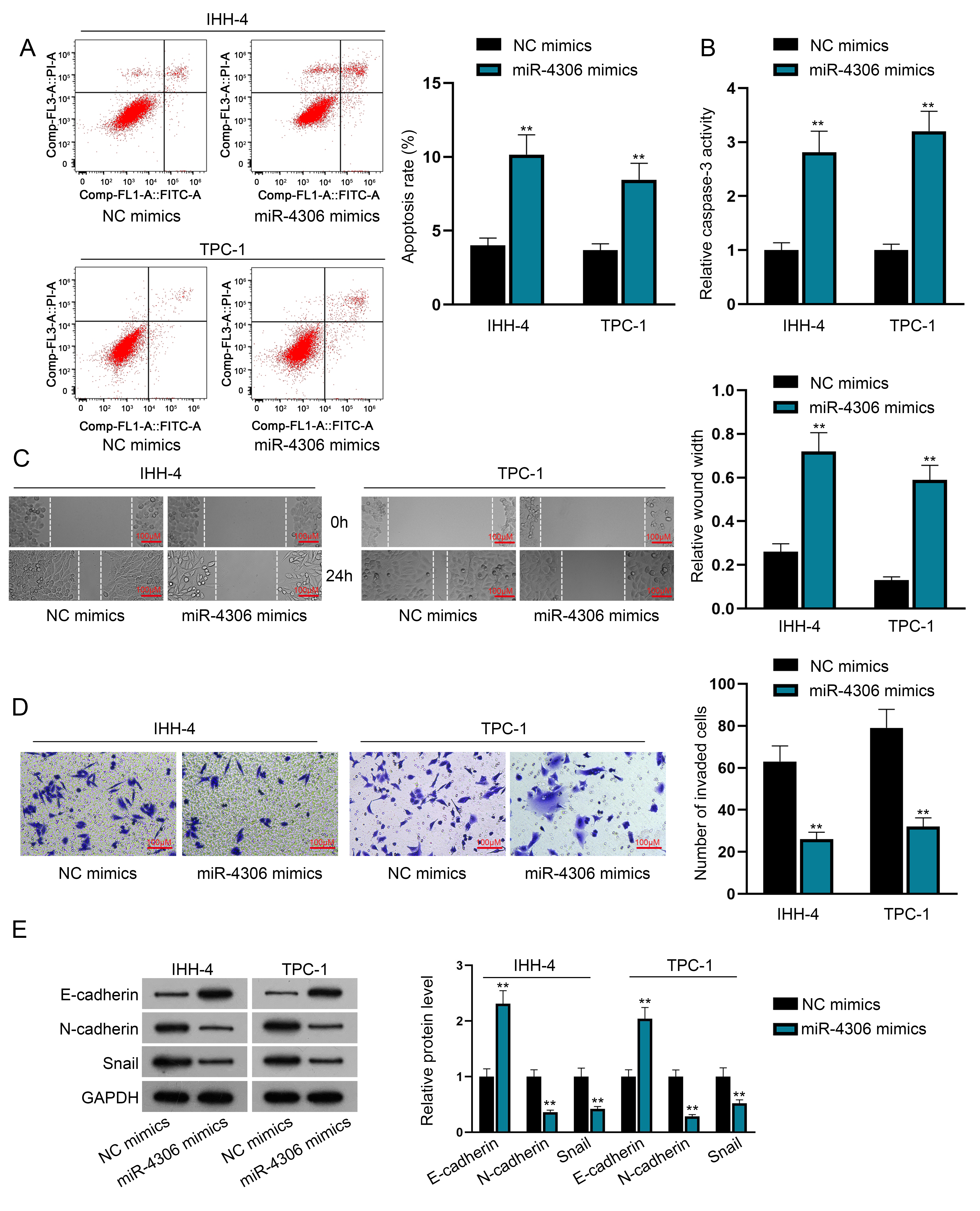

Supplement: Supplementary file 5 — Additional file 5: Figure S4 Upregulation of miR-4306 induces cell apoptosis and suppresses cell migration and invasion. A-B. The increased apoptosis induced by miR-4306 overexpression was observed through flow cytometry analysis and caspase 3 activity test. C-D. The inhibitory effects of miR-4306 mimics on the migration and invasion of PTC cells were also determined in accordance with the results of wound healing assay and transwell assay. E. Western blot assay was performed to detect the protein levels of EMT markers after the overexpression of miR-4306. **P < 0.01. [file 12935_2021_1950_MOESM5_ESM.tif]

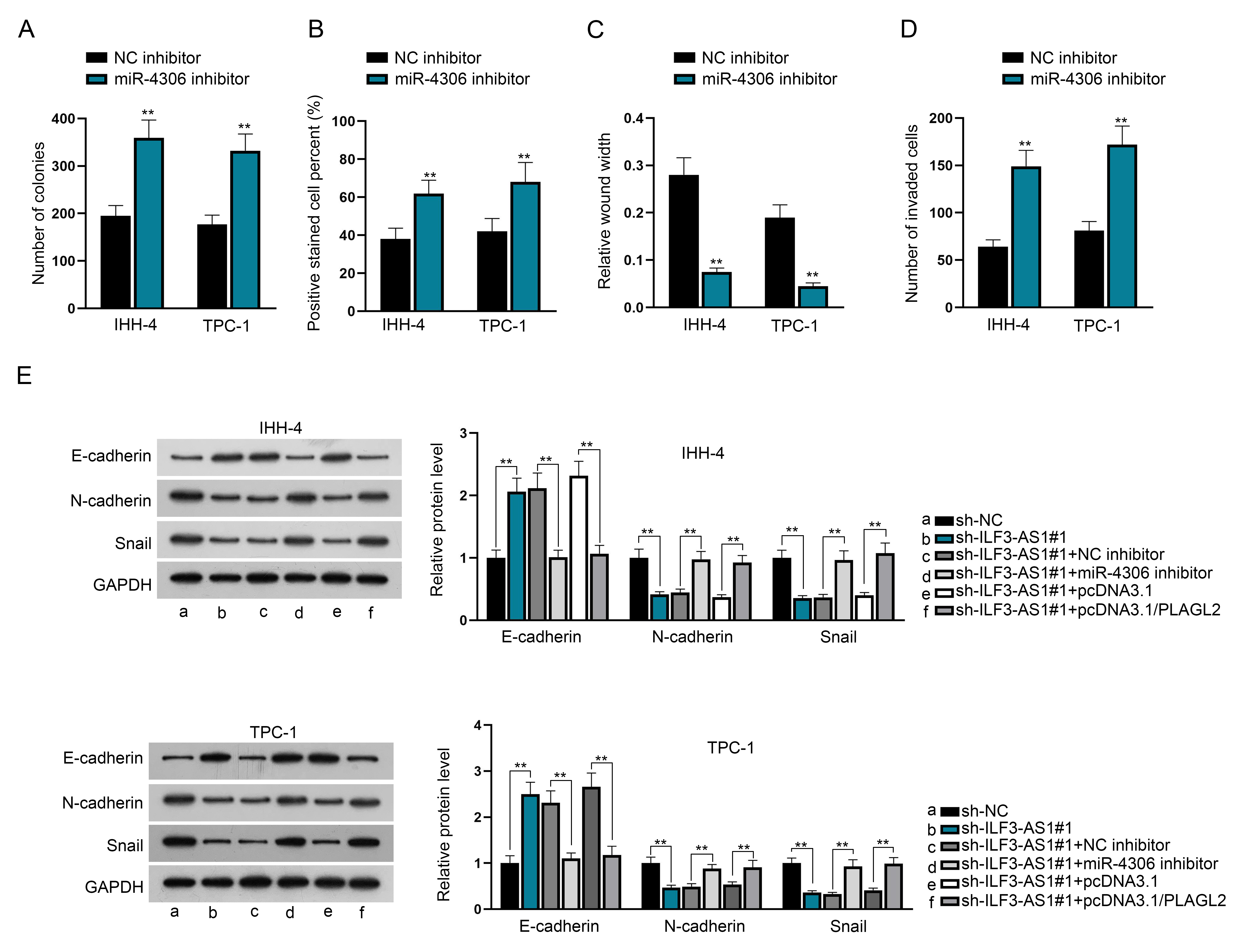

Supplement: Supplementary file 6 — Additional file 6: Figure S5 miR-4306 inhibition facilitates PTC cell growth and migration. A-B. Colony formation and EdU assays probed into the effects of miR-4306 inhibition on PTC cell proliferation. C-D. The effects of miR-4306 silencing on the capacities of PTC cells to migrate and invade were also determined by wound healing assay and transwell assay. E. Western blot analysis was used to detect the protein levels of EMT markers in transfected IHH-4 and TPC-1 cells. **P < 0.01. [file 12935_2021_1950_MOESM6_ESM.tif]
